# RNA Sequencing Reveals a Slow to Fast Muscle Fiber Type Transition after Olanzapine Infusion in Rats

**DOI:** 10.1371/journal.pone.0123966

**Published:** 2015-04-20

**Authors:** Christopher J. Lynch, Yuping Xu, Andras Hajnal, Anna C. Salzberg, Yuka Imamura Kawasawa

**Affiliations:** 1 Department of Cellular and Molecular Physiology, College of Medicine, Penn State University, Hershey, Pennsylvania, 17033, United States of America; 2 Department of Neural and Behavioral Sciences, College of Medicine, Penn State University, Hershey, Pennsylvania, 17033, United States of America; 3 Department of Public Health Sciences, College of Medicine, Penn State University, Hershey, Pennsylvania, 17033, United States of America; 4 Department of Pharmacology, College of Medicine, Penn State University, Hershey, Pennsylvania, 17033, United States of America; 5 Department of Biochemistry and Molecular Biology, College of Medicine, Penn State University, Hershey, Pennsylvania, 17033, United States of America; 6 The Institute for Personalized Medicine, College of Medicine, Penn State University, Hershey, Pennsylvania, 17033, United States of America; Universidad Pablo de Olavide, Centro Andaluz de Biología del Desarrollo-CSIC, SPAIN

## Abstract

Second generation antipsychotics (SGAs), like olanzapine, exhibit acute metabolic side effects leading to metabolic inflexibility, hyperglycemia, adiposity and diabetes. Understanding how SGAs affect the skeletal muscle transcriptome could elucidate approaches for mitigating these side effects. Male Sprague-Dawley rats were infused intravenously with vehicle or olanzapine for 24h using a dose leading to a mild hyperglycemia. RNA-Seq was performed on gastrocnemius muscle, followed by alignment of the data with the Rat Genome Assembly 5.0. Olanzapine altered expression of 1347 out of 26407 genes. Genes encoding skeletal muscle fiber-type specific sarcomeric, ion channel, glycolytic, O_2-_ and Ca^2+^-handling, TCA cycle, vascularization and lipid oxidation proteins and pathways, along with NADH shuttles and LDH isoforms were affected. Bioinformatics analyses indicate that olanzapine decreased the expression of slower and more oxidative fiber type genes (e.g., type 1), while up regulating those for the most glycolytic and least metabolically flexible, fast twitch fiber type, IIb. Protein turnover genes, necessary to bring about transition, were also up regulated. Potential upstream regulators were also identified. Olanzapine appears to be rapidly affecting the muscle transcriptome to bring about a change to a fast-glycolytic fiber type. Such fiber types are more susceptible than slow muscle to atrophy, and such transitions are observed in chronic metabolic diseases. Thus these effects could contribute to the altered body composition and metabolic disease olanzapine causes. A potential interventional strategy is implicated because aerobic exercise, in contrast to resistance exercise, can oppose such slow to fast fiber transitions.

## Introduction

Second generation antipsychotics (SGAs, also called, atypical antipsychotics), like olanzapine, are used to treat schizophrenia and bipolar disorder [1]. Owing to superior efficacy for psychiatric disorders compared to first generation drugs and a growing number of off- and on-label indications, the rate of new antipsychotic prescriptions has been growing steadily for adults and children [2]. For example, 2011 estimates indicated 3.1 million Americans were prescribed antipsychotics, up 13% from the year before [3]. Unfortunately, some SGAs cause adverse metabolic side effects that can negatively impact patient compliance. These side effects include hyperglycemia, glucose intolerance, weight gain, dyslipidemia, obesity, hyperglycemia, type 2 diabetes (T2D), ketoacidosis and even death [4–10]. Olanzapine and clozapine are two examples that may cause average body weight gains in the range to 1 pound per week [11].

While weight gain contributes to the metabolic complications of SGAs, there is strong evidence of body weight independent metabolic effects preceding obesity and type 2 diabetes. For example, dyslipidemia, hyperglycemia and glucose intolerance can be observed minutes or days after SGA administration, associated with rapid changes in muscle metabolism [12]. Endocrine-metabolic effects observed in plasma are sustained for weeks in healthy subjects and high fidelity preclinical models, prior to alterations in body composition/weight gain and increased hunger that appear later [11, 13–16]. Therefore understanding acute actions of SGAs might elucidate mechanisms contributing to these side effects.

Skeletal muscle is one of the most important tissues by weight for the disposal of glucose and macronutrients. As a major component of whole body energy balance it has the potential to impact the acute effects of SGAs on glycemia and glucose tolerance. Chronic olanzapine treatment in rats and dogs increased adiposity but not body weight [17–19]. This implies that changes in lean mass could be occurring, conceivably altering muscle energy requirements, susceptibility to atrophy and glycemia. Such changes could facilitate the adiposity elicited by olanzapine [17–19]. More information on how SGAs affects skeletal muscle is needed.

Skeletal muscles exhibit different fiber types with distinct features and energy requirements. While various classification schemes a simple classification is slow (oxidative, SO), intermediate (fast oxidative-glycolytic, FOG) and fast twitch (glycolytic, FG). SO fibers are also called type I, while the fastest-twitch glycolytic fibers (FG) in rats are type IIb. SO fibers contract or twitch more slowly than other types, are more efficient using oxygen to sustain contractions owing to their higher concentration of myoglobin (contributing to darker color), mitochondria and increased vascularity [20, 21]. They express myosin heavy chain 7 (*MYH7*, as found in soleus). At other end of the spectrum, FG fibers (IIb containing *MYH4*) have a very fast twitch force and very high power, but a low capillary density and mitochondrial and myoglobin content reflected in their whiter color [20–22]. Consequently, IIB fibers are less able to oxidize glucose or FFA, and instead, generate more ATP from anaerobic glycolysis. FOG fiber types exhibit intermediate characteristics and unique myosin heavy chains (e.g., *MYH2* and *MYH1*) and can be further sub-divided. An important difference between slower and faster fibers, in addition to energy consumption and twitch speed, is that FG fibers are more susceptible than SO to atrophy arising from various metabolic pathologies [23]. In obesity and T2D, skeletal muscle has been associated with either a loss of oxidative enzyme capacity irrespective of muscle fiber type [24], or a transition of muscle fiber types from more SO to the less metabolically flexible FG fibers [25–29].

Given the importance of skeletal muscle as a major consumer of whole body energy and macronutrients, it is important to understand how olanzapine affects this tissue before significant changes in adiposity occur. Therefore in this study we used RNA-Seq to quantify gene expression in response to continuous infusion of olanzapine for 24h using a protocol that produces a mild hyperglycemia.

## Materials and Methods

### Ethics Statement

All of the vertebrate animal procedures were approved by the Institutional Animal Care and Use Committee (IACUC) of Penn State University College of Medicine (Hershey, PA). The Animal Resource Program is accredited by AAALAC International. All animal living conditions are consistent with standards laid forth in the Guide for the Care and Use of Laboratory Animals (2011), 8th edition, published by the National Research Council.

### Olanzapine infusion

Male Sprague Dawley rats (150–175g) from Charles River Laboratories (Cambridge, MA, USA) were acclimated to single housing for several weeks while being maintained on a 12:12 h light dark cycle at ~21°C and provided free access to water and a standard rodent chow (2018, Harlan Laboratories, Madison, WI). Under brief isoflurane anesthesia, venous (jugular) and arterial (carotid) catheters were surgically implanted as previously described [12, 18] and an external neck collar was placed for use in the CMA/120 infusion system (CMA7431059, Harvard Apparatus, Hollison, MA). A week or more after this surgery, all the experimental animals had regained their body weight trajectories. The night before infusions, rats were individually housed in a CMA/120 Rat System for Freely Moving Animals. They continued to have free access to the same food, water and dried corncob bedding used in the home cages. The next day catheters were connected to the catheter swivel system. Heparinized glucose determinations were obtained as described [12, 18], prior to the start of the infusions study ~2PM. For the olanzapine group, the venous catheter was attached to a New Era Pump Systems syringe pump (Model NE-300) filled with olanzapine (Dr. Reddy’s Laboratories Ltd, Hyderabad, India) in sterile saline (infusion: 1mg/100g BW loading dose for 0.5h and then 0.04mg/100g/h continuously for 23.5h). Control rats were infused with vehicle at the same rate and volume. At the conclusion of the infusions additional heparinized plasma and blood glucose determinations were obtained for blood glucose determination. Gastrocnemius was then surgically removed under isoflurane anesthesia (carried with 100% O_2_), and frozen between two aluminum blocks cooled to the temperature of liquid nitrogen and then stored at -80°C until RNA was isolated. While under continuous anesthesia, the animals were euthanized by cutting the diaphragm and removing the heart.

### RNA Sequencing

Total RNA was extracted as previously described with slight modification [30]. Briefly, frozen muscle tissue was pulverized in liquid nitrogen using mortar and pestle, followed by bead mill homogenization (Bullet Blender, Next Advance) using stainless steel beads (Next Advance, cat# SSB14B) and mirVana RNA isolation kit (Life Technologies). Optical density values of extracted RNA were measured using NanoDrop (Thermo Scientific) to confirm an A_260_:A_280_ ratio above 1.9. RIN was determined for each sample using Bioanalyzer RNA 6000 Nano Kit (Agilent Technologies). The cDNA libraries were prepared using the TruSeq RNA Sample Prep Kit v2 (Illumina) as per the manufacturer’s instructions. The final product was assessed for its size distribution using Bioanalyzer DNA High Sensitivity Kit (Agilent Technologies) and for its concentration using Kapa library quantification kit (Kapa Biosystems). Six libraries were pooled per HiSeq lane, followed by on-board cluster generation on a Rapid Run single-end flow cell and subsequent 50 cycles sequencing (v3 sequencing kit) according to the manufacturer's instructions (HiSeq 2500, Illumina). Demultiplexed and quality filtered mRNA-seq reads were then aligned to Rat reference assembly (Rn5) using TopHat (v.2.0.9). The uniquely mapped reads were used to calculate the normalized expression level of genes, as fragments per kilobase of exon per million fragments mapped (FPKM), using Cufflinks (v.2.2.1).

### Quantitative Real-Time-PCR (QT-RTPCR)

Equal quantities of total RNA were used to synthesize cDNA using the High Capacity cDNA RT Kit with RNase Inhibitors (Applied Biosystems, Inc.). For QRT-PCR reactions, the TaqMan Gene Expression Assays (Applied Biosystems, Inc.) specific for rat *Eef2*, *OSTN*, *Casq2*, *LPL*, *Alp2a2*, *Tnni1*, *Pavlb*, and *TPM1* was performed according to the manufacturer's instructions and was carried out with equal quantities of cDNA. The assays were run on a 7900HT PCR system (Applied Biosystems, Inc.) available at the College of Medicine Core Lab and were analyzed using the comparative Ct method. The results were normalized to internal *Eef2* mRNA controls.

### Biostatistics and Bioinformatic Analysis

To determine significant differences in mean FPKM values between control and olanzapine groups, the DEGexp function of the DEGseq 1.18.0 R package was used with the Likelihood Ratio Test (LRT) and default parameters. A p<0.05 was used to determine significant differences, however genes where no detectable RNA was observed in some subjects were flagged (#VALUE!) and not considered further. Graphpad Prism 6.0 was used to examine statistical changes in QT-RTPCR data by Students t-test (p<0.05 was considered statistically significant) and linear regression analysis.

Functional annotation clustering, pathway analysis and gene ontology along with associated confidences indicating the strength of evidence for pathway effects were obtained using a series of tools including Ingenuity Pathway Analysis (IPA, Ingenuity Systems, www.ingenuity.com). Database for Annotation, Visualization and Integrated Discovery (DAVID, http://david.abcc.ncifcrf.gov/), Kyoto Encyclopedia of Genes and Genomes (KEGG, www.kegg.jp), GeneCards (www.genecards.org) and the Rat Genome Database (RGD, http://rgd.mcw.edu/). Pathway analysis was performed on genes that were statistically different based on DEGSeq analysis. Olanzapine rapidly elevates plasma glucocorticoids as it does glucose [31]. The potential impact of glucocorticoid changes on gene expression and needs to be considered in interpreting the findings [31]. Genes affected by this increase in corticosteroid concentrations were either identified from the above pathway analysis packages or by examining microarray time course data from muscle tissue during constant infusion of methylprednisolone in adrenalectomized rats (NCBI Geo profile GDS2688) [32].

### Data Sharing

The processed data (S1 Table), associated metadata, and the “raw” Illumina fastq file have been deposited with the National Center for Biotechnology Information (NCBI) Gene Expression Omnibus database (GEO Accession number: GSE65787).

## Results and Discussion

Rats with comparable body weights (vehicle: 373±9g, olanzapine: 388±11g, p = 0.34) were infused with vehicle (control) or olanzapine for 24h. The olanzapine infusion produced a modest 30% increase in blood glucose (vehicle, 98±2mg/dl; olanzapine 127±4mg/dl, p = 0.0023). This acute effect is consistent with previous findings in humans and animal models, however far greater rises in blood glucose can be achieved with higher doses [31]. Transcriptome sequencing of mRNA of gastrocnemius muscle from rats in the control group resulted in FPKM values expected for striated skeletal muscle tissue. For example, the top one hundred FPKM values were associated with genes that encode proteins of the skeletal muscle contractile machinery, cation transporters, glycolysis, oxidative metabolism and protein synthesis (S1 Table).

Cluster analysis indicated that the data from each rat clustered into their respective control or OLZ treatment groups (not shown). Of 26,406 genes in the rat genome build we used, 1451 genes were determined to be significantly different due to olanzapine (S1 Table and S2 Table). However 104 of the p<0.05 genes were not studied further because the data from DEGexp were flagged (“#Value!”, S2 Table) due to no detectable counts in one or more animal (S1 Table).

**Fig 1:**
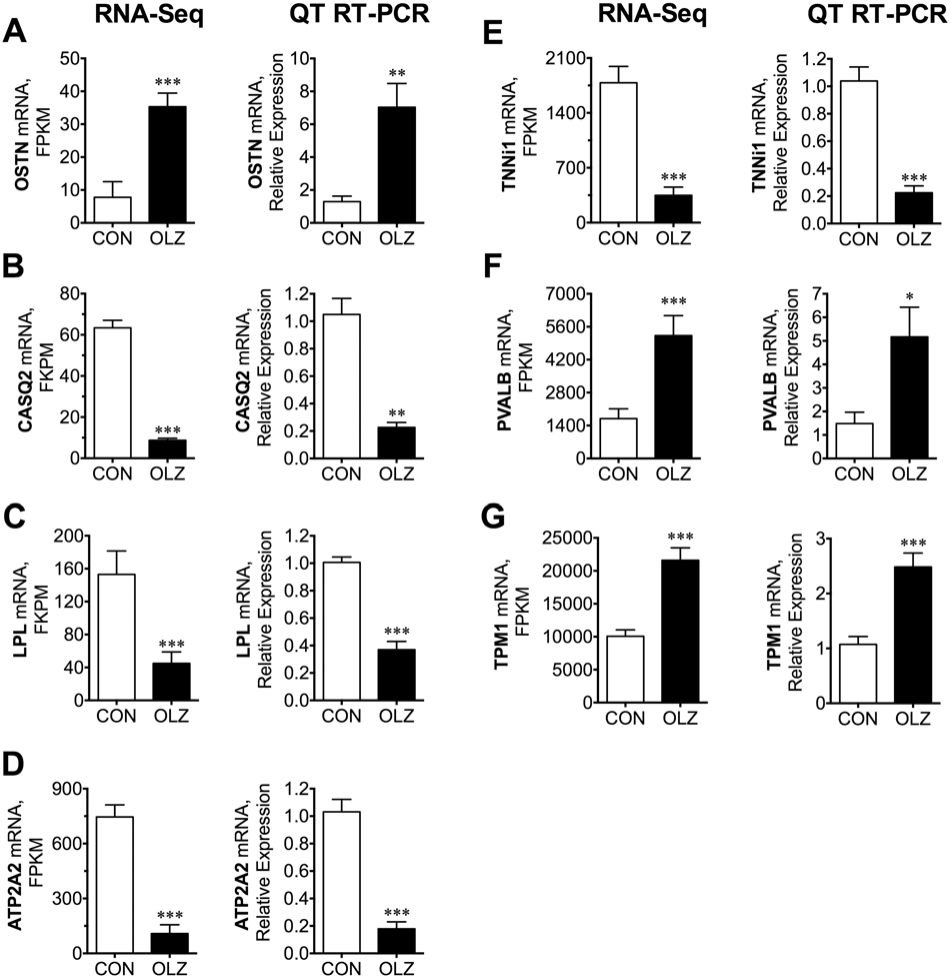
Comparison of RNA-Seq to QT-RTPCR for selected gastrocnemius muscle genes affected by olanzapine infusion. Genes significantly affected by olanzapine infusion based on RNA-Seq were selected for QT-RTPCR analysis using TaqMan gene expression assays using the same preparation of RNA. Genes were selected so that different FKPM size bins (1–10: *OSTN*; 10–99: *OSTN*, *Casq2*, *Lpl*; 100–999: *Lpl*, *Atp2a2*, *Tnni1*, 1000–9000: Tnni, Pvalb; >9000: Tpm1) were represented. The bars show mean± SE. Statistical significance was determined using the DEGSeq R package, which takes into consideration the variability of all of the genes analyzed. QT-RTPCR differences were analyzed using Student’s t-test. Asterisk symbols indicate a significant difference compared to control, *: p <0.05, **: p<0.01, ***: p<0.001)

We next sought to compare genes with statistically different changes due to OLZ according to DEG-Seq to QT-RTPCR data. Our goal was to examine compare these two over different FKPM bin ranges (e.g., with one value below 10,10–100, 100–1000, 1000–10,000 and >10,000) compared to a QT-RTPCR (Fig 1). We endeavored to have at least one OLZ and one Control value in different bins so that more bins could be evaluated. *Ostn*, *Pvalb and Tpm1* expression was significantly elevated by olanzapine treatment based on RNA-Seq and exhibited close proportionally elevated changes based on QT-RTPCR assays using *Eef2* expression as a normalizer. Consistently, olanzapine similarly decreased the expression of *Tnni1*, *Casq2*, *Lpl* and *Atpa2* as determined by either RNA-Seq or QT-RTPCR. The effects of olanzapine based on these two methods were highly correlated (r2 = 0.98, S1 Fig). Thus, these findings suggested that our RNA-Seq data were similarly quantitative as QT-RTPCR, albeit the QT-RTPCR data generally exhibited comparatively higher normalized standard deviations (not shown) as reflected in the size of the standard errors (Fig 1). A weakness is that we did not study QT-RTPCR of genes with lower % FKPM changes, however our conclusions in the results below about affected pathways are largely based on changes in many rather than individual genes. Furthermore, other studies have demonstrated that RNA-Seq is highly accurate for quantifying expression levels [33].

### Olanzapine alters skeletal muscle fiber type genes

Bioinformatic analysis of the RNA-Seq data indicates that olanzapine is causing a muscle fiber type transition to the most glycolytic and fastest twitch fiber type (IIb). Multiple lines of evidence support this. The first is an observed decrease in the genes for a number of myosin heavy chain genes and an increase in myosin heavy chain 4, *Myh4* (Table 1). The second is altered expression of other genes and isoforms that comprise fiber type specific components of the muscle sarcomere and muscle cation transporters (Table 1). The categorization of these genes as slow or fast is based on literature along with annotation from the RGD and Genecard databases. Based on these sources, FG fibers (e.g., IIb) normally express higher levels of Actin 3 (*Actn3*), Na^+^/K^+^-ATPase subunit isoform 2B (*Atp1b2)*, Serca1 (ATPase, Ca^2+^ transporting, cardiac muscle, fast twitch 1;a.k.a., *Atp1a1*), calsequestrin 1 (fast-twitch, skeletal muscle; *Casq1*), myosin, light chain 1, alkali; skeletal, fast (*Myl1*), myosin light chain, (phosphorylatable, fast skeletal muscle; *Mylpf*), myosin binding protein C, fast type (Mybpc2), myomesin 2 (a.k.a. M-protein; *Myom2*), myozenin 1 (a.k.a. calsarcin 2; *Myoz1*), parvalbumin (*Pvalb*), troponin C type 2 (fast;*Tnnc2*), fast skeletal muscle troponin I (*Tnni2*), fast skeletal muscle troponin T gene (*Tnnt3*) and fast twitch tropomyosin 1 (*Tpm1*) [34–48].

**Table 1:** Olanzapine shifts expression of genes associated with skeletal muscle fiber type.

Fast twitch/ type IIb genes				Slow twitch or intermediate fiber type genes			
Gene name	Vehicle, FPKM	Olanzapine, FPKM	Normalized fold change	Gene Name	Vehicle, FPKM	Olanzapine, FPKM	Normalized fold change
*Actn3*	2145±503	3194±379	+1.4	*Actn2*	1259±5	451±60	-2.9
*Atp1b2* *	105±26	162±4.3	+1.5	*Atp1b1* * *Atp1a1*	104±21 34±1.3	32±6 23±4.0	-3.4 -1.5
*Atp2a1*	7138±811	8284±766	+1.2	*Atp2a2* *	793±71	115±48	-7.1
*Casq1*	1307±218	2295±307	+1.8	*Casq2*	65±4.5	9.2±1.1	-7.3
				*Csrp3*	419±87	84±16	-5.2
*Fhl3*	167±31	328±63	+2.0	*Fhl1*	1913±20	491±98	-4.0
				*Mb*	11791±2736	7193±1471	-1.7
*Mybpc2*	844±113	1208±149	+1.4	*Mybpc1*	1024±63	469±47	-2.3
*Mybph*	114±41	229±71	+1.9				
*Myh4* (type IIb)	1644±612	2998±373	+1.8	*Myh1* (IIx)	1245±117	787±52	-1.6
				*Myh2* (IIa)	361±57	790±24	-4.2
				*Myh3*	8.8±2.2	2.2±0.7	-8.6
				*Myh6*	11±2.4	2.7±0.2	-4.3
				*Myh7* (I)	653±59	88±44	-7.7
				*Myh7b*	4.4±0.6	0.66±0.28	-6.9
				*Myh8*	9.9±2.3	5.3±0.3	-1.9
				*Myh10* *	9.2±0.7	1.4±0.2	-6.6
*Myl1*	12213±951	19839±1339	+1.6	*Myl2*	12267±2974	2364±583	-5.4
*Mylpf*	15708±1019	38303±5017	+2.4	*Myl3*	3398±602	708±153	-5.0
				*Myl6b*	312±43	65±32	-5.0
				*Myl12a*	389±55	215±22	-1.9
*Myom2*	270±46	256±32	N.S.	*Myom3*	42±3.4	7.1±4.0	-7.2
				*Myom1*	144±9	114±10	-1.3
				*Myog*	25±2	12±0.6	-2.3
*Myoz1*	1354±187	2426±345	+1.8	*Myoz2*	571±83	115±42	-5.1
*Pvalb*	1778±450	5568±958	+3.0				
				*Obscn*	93±18	71±6	-1.4
*Tnnc2*	8963±518	14528±1422	+1.6	*Tnnc1*	2129±331	486±176	-4.6
*Tnni2*	9604±504	17974±2203	+1.8	*Tnni1*	1595±181	313±93	-5.3
*Tnnt3*	12592±882	18845±2840	+1.5	*Tnnt1*	2020±380	309±121	-6.8
*Tpm1*	7939±828	16186±1342	+2.0	*Tpm2*	5455±74	3561±74	-1.6
				*Tpm3*	1218±186	215±81	-5.9

Isoforms of fast and slow twitch muscle fiber genes with statistically significant changes when comparing Vehicle and Olanzapine groups are indicated by the presence of a normalized fold change value. Data are means plus or minus (±) SE of the fragments per kilobase of exon per million fragments mapped values (FKPM). N.S. indicates no significant difference. An asterisk(*) indicates a prioritized T2D or obesity candidate gene [49].

In contradistinction to FG fibers, SO fibers express more Actin 2 (*Actn2*), *Atp1a1*, *Atp1b1* (isoforms of Na^+^/K^+^-ATPase subunits), *Atp2a2 (also called Serca2*: ATPase, Ca^++^ Transporting, Cardiac Muscle, Slow Twitch 2), calsequestrin 2 (*Casq2*), Four-And-A-Half LIM Domains 1 (a.k.a. SLIM1, *Fhl1*), myoglobin (*MB*), myosin binding protein C, slow type (*Mybpc1*), myosin, light chain 2, regulatory, cardiac, slow (*Myl2*), myosin, light chain 3, alkali; ventricular, skeletal, slow (*Myl3*), myomesin 3 (*Myom3*), myogenin (*Myog*), myozenin 2 (a.k.a. calsarcin-1, *Myoz2*), obscurin (*Obscn*), troponin C type 1, slow (*Tnnc1*), troponin I type 1,skeletal, slow (*Tnni1*), tropomyosin 2 beta (*TPM2*) and tropomyosin 3 (*Tpm3*) genes [35, 40, 42, 46, 47, 50–54]. Thus, olanzapine infusion reduced the expression of genes encoding contractile and cation transport isoforms found in slow or less glycolytic fast fibers (type 1 & non-IIb, a.k.a., SO & FOG) and up regulated those coding for the most glycolytic type of the fast fibers, type IIb (Table 1). Notably, two of the affected Na^+^/K^+^-ATPase subunits along with the calcium ATPase, Serca2, have been designated as obesity or T2D candidate genes [49]. Olanzapine also decreased the expression of the gene coding for Muscle LIM protein (*CRP3*, *Csrp3*) which is involved in slow myosin heavy chain expression and fast to slow fiber transitions [55]. While adult rat gastrocnemius is generally considered a mixed muscle, its fiber composition already contains a majority of FG fibers [56]. That explains why the magnitude of the change in IIb-related genes was generally less than the magnitude of lowering of slower twitch fiber associated genes.

### Metabolic gene expression in skeletal muscle after olanzapine

**Fig 2:**
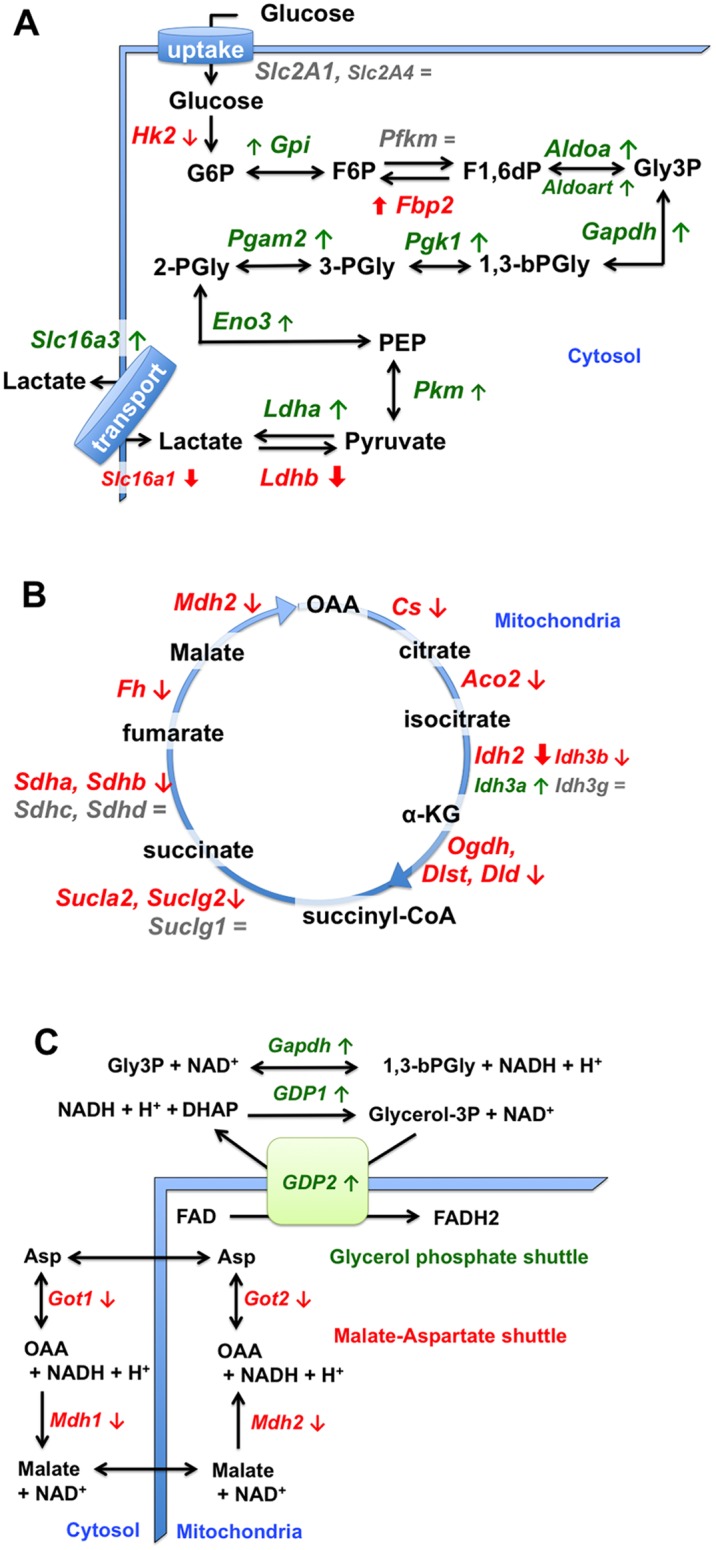
Changes in glycolytic, TCA cycle and mitochondrial shuttle genes in gastrocnemius muscle after olanzapine infusion. Effects of olanzapine infusion on muscle gene expression are shown for the glycolysis pathway (A), citric acid (TCA) cycle (B) and two mitochondrial shuttles (C; the mitochondrial malate, *SLC25A11*, and aspartate, *SLC25A13*, transporters were not affected). The blue lines indicate the sarcolemma (A) or the mitochondrial membrane (C). Metabolites and glycolytic intermediates are shown in black font; gene names are *italicized*. Arrows indicate an increase (↑,**⬆**) or decrease (↓,**⬇**) in gene expression due to olanzapine infusion; bold arrows indicate a larger magnitude of change. Gray font and equal sign (=) indicates no significant change. Colored font indicates a change predicted to oppose (red) or promote (green) the pathway. Where the abundance of two isoforms is different, the font size is smaller for the isoform where the FPKM has a lower value (S3 Table).

**Fig 3:**
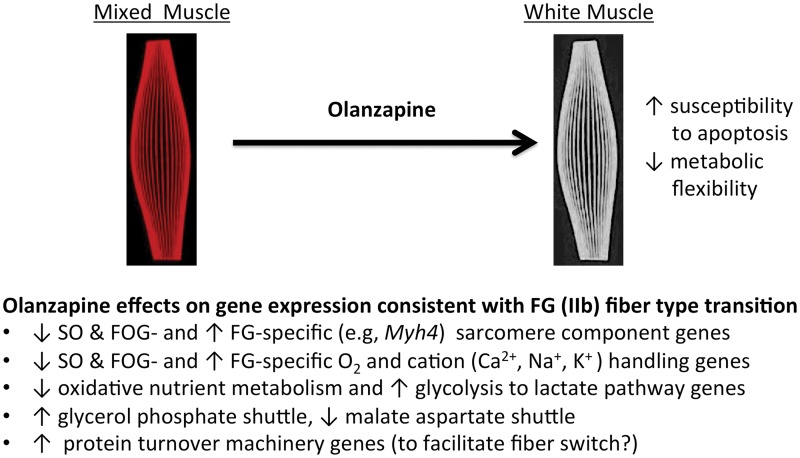
Simplified schematic summarization of muscle fiber types and the pathways affected by olanzapine infusion in skeletal muscle. Striated skeletal muscle fibers can be categorized into three or more different types such as (1) slow-twitch oxidative (SO, red), type I, (2) fast-twitch oxidative-glycolytic (FOG, intermediate twitch) and in rats (3) the fastest-twitch glycolytic (FG, white), type IIb. Gastrocnemius, the muscle examined in this paper, normally contains all of these. Fiber types differ in twitch speed and metabolic flexibility. They are frequently categorized into the above types based one or more of the following: myosin heavy chain isoform content or myosin ATPase activity or gene expression. Compared to SO fibers, FG fibers have: (a) fewer mitochondria, reduced vascularity and myoglobin (*Mb*) for O_2_ handling making them whiter in appearance compared to the redder SO fibers; (b) lower expression of genes in FFA, glucose and amino acid oxidation pathways, (c) increased expression of most genes in the glycolysis to lactate pathway; (d) different NADH shuttles; (e) fiber type specific expression of specific sarcomere components, and (f) specific isoforms of calcium and monovalent cation handling or transport proteins. Our data suggest that acute exposure to olanzapine is beginning a process that will eventually cause a fiber type transition from a mixed type to a whiter FG (IIb) type. Whiter muscle has been reported to be more susceptible than other fiber types to atrophy, and such fiber type transitions changes are associated with metabolic disease and obesity.

Pathway analyses revealed significant changes in glycolysis (p = 1.3E-7), the citrate cycle (TCA cycle, p = 7.7e-14), fatty acid metabolic processes (p = 6.4e-7), and oxidative phosphorylation (p = 1.8e-38) including fatty acid and branched chain amino acid oxidation (p = 1.8e-7). Consistent with a slower to faster fiber type transition, olanzapine increased the expression of most genes in glycolysis (Fig 2A, S3 Table). However, a rate-controlling step in glycolysis, catalyzed by the skeletal muscle isoform of hexokinase (*HK2*), decreased -1.9 fold (Fig 2A, S3 Table). This may be related to the observation that muscle hexokinase, *HK2*, is expressed at higher levels in slower compared to more glycolytic fibers, e.g.: [57]. Notably, muscle hexokinase activity is also depressed in insulin resistance and diabetes [58, 59]. Therefore changes in HK2 are also consistent with the concept of a slow to fast twitch fiber type transition and olanzapine’s effect on (Fig 3). FG fibers are more likely to convert pyruvate from glycolysis to lactate and export the lactate rather than oxidizing the pyruvate in mitochondria. Thus consistent with a fiber type transition being underway, olanzapine increased the expression of *Ldha* and *Slc16a3* normally involved in non-oxidative glucose metabolism and cellular lactate efflux, and decreased expression of the genes coding isoforms of lactate dehydrogenase and lactate transporter that have been associated with increased lactate influx and metabolism, *Ldhb and Slc16a1* (also called *MCT1*, see Fig 2A) [60]. Creatine kinase gene expression (*CKM*) was also increased by olanzapine (S1 Table and S2 Table), this too is associated with fiber type switching and anaerobic ATP formation in T2D [61].

In contradistinction to the up-regulation of glycolysis, but consistent with an SO/FOG to FG transition, olanzapine depressed the expression of most TCA cycle genes (Fig 2B and S3 Table). An exception was with isocitrate dehydrogenase (IDH) subunit genes. *Idh3b*, *Idh3a* and *Idh3g* showed inconsistent changes or no significant difference, whereas *Idh3b*, an obesity candidate gene [49], was decreased, as was another IDH (*Idh2*, -2.3 fold) that is highly expressed in gastrocnemius (Fig 2B, S3 Table). However of the genes associated with the pyruvate dehydrogenase complex’s entry point into the TCA cycle, only Pdhb and Dld were decreased (S1 Table and S2 Table).

Olanzapine shifted expression of the mitochondrial NAD^+^/NADH shuttles used to regenerate NAD^+^ for glycolysis (Fig 2C, S1 Table). Genes for the malate aspartate shuttle were decreased, whereas those for the glycerol phosphate shuttle were increased. The observed decrease in malate-aspartate shuttle is consistent with a the concept of a transition being underway to a less oxidative muscle fiber type [62].

Further consistent with a shift to a more glycolytic fiber type, sarcolemmal, cytosolic and mitochondrial genes coding for enzymes and transporters in lipid oxidation were reduced by olanzapine (Table 2). For example, *Lpl* that encodes lipoprotein lipase was -3.5 fold lower. Similarly, gene expression of the sarcolemmal fat transporters, FAT (*Slc27a1*) and fatty acid translocase (*Cd36)* were reduced along with the cytosolic muscle fatty acid binding protein (Fabp3) and enzymes that convert fatty acids to their respective acyl-CoAs were reduced, along with genes for fatty acyl-CoA mitochondrial transport and oxidation, including the rate-controlling step in muscle fatty acid oxidation (*Cpt1b*, Table 2). In contradistinction, olanzapine infusion increased the expression the mitochondrial fatty acid transporter gene, *UCP3*, and that of the adipose tissue specific fatty acid binding protein 4 (*FABP4*, also called *AP2*, Table 2 and S1 Table). While we have no explanation for these changes, it is noted that increased skeletal muscle *UCP3* expression occurs in T2D and during slow to fast twitch muscle fiber type transitions [63, 64]. Furthermore, *FABP4* is the adipose/macrophage fatty acid binding protein, not the muscle specific fatty acid binding protein, FABP3, which did decline (Table 2 and S1 Table). The rise in FABP4 could potentially harken an increase in macrophage presence/inflammation in skeletal muscle and/or an increase in muscle associated adipose tissue. Further studies would be needed to examine these ideas.

**Table 2:** Statistically significant effects of olanzapine on the expression of genes involved in oxidative lipid metabolism.

Gene name	Vehicle, FPKM^1^	Olanzapine, FPKM^1^	Normalized fold change	Molecular identity
*Acaa2* *	233±40	145±8	-1.7	Acetyl-CoA acyltransferase 2
*Acad9*	31±5	14±2	-2.2	Acyl-CoA dehydrogenase family, member 9
*Acadl*	384±72	221±8	-1.8	Acyl-CoA dehydrogenase, long chain
*Acadm*	426±47	245±18	-1.8	Acyl-CoA dehydrogenase, C-4 to C-12 straight chain
*Acads* *	102±13	78±8	-1.4	Acyl-CoA dehydrogenase, C-2 to C-3 short chain
*Acadsb*	31±7	18±2	-1.8	Acyl-CoA dehydrogenase, short/ branched chain
*Acat1*	285±45	210±19	-1.4	Acetyl-CoA acetyltransferase, mitochondrial also known as acetoacetyl-CoA thiolase
*Acadvl* *	163±16	101±5	-1.7	Acyl-CoA dehydrogenase, very long chain
*Acox1*	24±1	16±2	-1.6	Acyl-CoA oxidase 1, palmitoyl
*Acsl1*	139±28	73±12	-2.0	Ayl-CoA synthetase long-chain family member 1
*Acss1*	20±3	4.7±0.7	-4.5	Acetyl-CoA synthetase, mitochondrial
*Acss2*	19±4	12±0.1	-1.7	Acetyl-coenzyme A synthetase, cytoplasmic
*Alox15*	9.1±7.0	1.9±0.5	-2.2	Arachidonate 15-lipoxygenase
*Cd36*	40±8	24±2	-1.7	Fatty acid translocase (transmembrane)
*Cpt1b*	131±11	91±6	-1.5	Carnitine palmitoyltransferase 1b, muscle
*Cpt2*	67±11	40±3	-1.7	Carnitine palmitoyltransferase 2
*Eci1*	142±15	103±10	-1.4	Enoyl-CoA delta isomerase 1
*Ech1* *	277±32	236±4	-1.2	Enoyl Coenzyme A hydratase 1, peroxisomal
*Fabp3*	1386±251	633±68	-2.3	Fatty acid binding protein 3, muscle
*Fabp4*	277±25	383±90	+1.3	Fatty acid binding protein 4, adipocyte
*Hadh*	94±13	49±1	-2.0	Medium and short-chain L-3-hydroxyacyl-coenzyme A dehydrogenase, mitochondrial
*Hadha*	262±25	151±8	-1.8	Trifunctional protein, alpha subunit
*Hadhb*	447±75	328±3	-1.4	Trifunctional protein, beta subunit
*Lpl* *	174±31	51±15	-3.5	Lipoprotein Lipase
*Slc25a20*	84±11	52±2	-1.7	Carnitine/acylcarnitine translocase (cytosol mitochondrial)
*Slc27a1* *	11±1	4±1	-2.8	Solute carrier family 27 (Fatty acid transporter), Member 1
*UCP3*	23±7	44±18	+1.9	Uncoupling protein 3, mitochondrial

The role of these genes in lipid metabolism was determined from RGD, KEGG, Genecards and/or IPA websites as described in Methods. Results are mean ± SE of FPKM gene expression values or normalized fold changes found to be statistically significant using the DEGSeq R package. An asterisk (*) indicates one of 164 prioritized obesity or T2D candidate genes from a previous study [49]. The corresponding molecular identity and database number is in S1 Table.

Several genes responsible for mitochondrial BCAA oxidation [65] were decreased by olanzapine (S4 Table), including the catalytic subunit of branched chain keto acid dehydrogenase (*BCKDHA*) and the next step in leucine metabolism catalyzed by isovaleryl-CoA dehydrogenase (*IVD*). Both of these are obesity or diabetes candidate genes [49].

Consistent with the down regulation of genes in glucose, amino acid and fatty acid oxidation, IPA pathway analysis of gene expression indicated that a canonical pathway termed “Mitochondrial Dysfunction” was increased by olanzapine. While many of the genes in this pathway were only modestly affected, those effects were individually highly significant and when taken together as a pathway it was the most significantly affected process with P = 1.9e-46 (S5 Table). These 72 genes largely comprise mitochondrial and nuclear-encoded mitochondrial genes in the oxidative phosphorylation pathway, including complexes I–V.

To summarize, a third piece of evidence supporting a transition to a more glycolytic type (IIb) arises from the observation of a general increase in glycolytic genes and diminution in those for oxidation of glucose, lipid and branched chain amino acids, consistent with the known metabolic differences between muscle fiber types [56, 59, 66].

### Olanzapine effects on genes related to protein turnover

In order to transition from one fiber type to another, the sarcomeric and non-sarcomeric components specific to the slower fiber types would have to be degraded and replaced with new components found in IIb fibers. Gene expression and pathway analyses are consistent with this idea. Pathway analysis revealed increases in the EIF2 Signaling pathway for global protein synthesis and genes involved in apoptosis and proteosomal mediated degradation. Specifically, olanzapine up-regulated forty-nine genes (p = 1.9e-21) in the EIF-2 signaling-protein synthesis pathway, these include several dozen ribosomal protein genes (Table 3). Ingenuity pathway analysis predicted that apoptosis was also preparing to be activated (Z-score of 2.6) with 142 genes affected (p = 1.4e-16, data not shown). In addition, two of four key genes associated with proteolysis, catabolic disorders and atrophy in skeletal muscle, *TRIM63* (MuRF1) and *Ddit4l* (REDD2) [67, 68], were significantly up regulated (~1.4–1.5 norm FC), whereas two others, Ddit4 (REDD1) and Fbxo32 (atrogin-1) were not affected (S1 Table). Olanzapine also up-regulated the expression of genes involved in the 20S core particle of the proteosome including: *PSMB1*, *PSMB4*, *PSMB5*, *PSMB6*, *PSMB7*, *PSMA7*, along with genes for several ubiquitin-conjugating enzymes: *UBE2E2*, *UBE2G1*, *UBE2G2 and UBE2S* (S1 Table).

**Table 3:** Canonical EIF2-signaling pathway/ protein synthetic machinery genes statistically altered by olanzapine infusion in gastrocnemius.

Gene name	Vehicle, FPKM^1^	Olanzapine, FPKM^1^	Normalized fold change	Molecular Identity
*Akt2*	80±1	101±5	1.2	v-akt murine thymoma viral oncogene homolog 2
*Eif2s2*	50±6	73±6	1.4	eukaryotic translation initiation factor 2, subunit 2 beta, 38kDa
*Eif3i*	40±4	57±2	1.4	eukaryotic translation initiation factor 3, subunit I
*Eif4a2*	210±30	246±25	1.13	eukaryotic translation initiation factor 4A2
*Fau*	45±11	65±17	1.4	Finkel-Biskis-Reilly murine sarcoma virus (FBR-MuSV) ubiquitously expressed (chr1: 228355747–228357261)
*Hras1*	31±1	64±4	2.0	Harvey rat sarcoma viral oncogene homolog
*Ppp1ca*	137±15	187±21	1.3	protein phosphatase 1, catalytic subunit, alpha isozyme
*Ppp1cb*	133±18	119±8.5	-1.2	protein phosphatase 1, catalytic subunit, beta isozyme
*Rpl10*	185±23	233±30	1.2	ribosomal protein L10
*Rpl11*	317±28	429±57	1.3	ribosomal protein L11
*Rpl12*	0.6±0.3	10±4	18.6	ribosomal protein L12 (chr3:17346298–17348585)
*Rpl13a*	65±6	88±14	1.3	ribosomal protein L13a
*Rpl14*	190±17	254±25	1.3	ribosomal protein L14
*Rpl17*	26±12	38±19	1.4	ribosomal protein L17
*Rpl18*	202±13	262±26	1.3	ribosomal protein L18
*Rpl18a*	604±56	763±83	1.2	ribosomal protein L18a
*Rpl19*	190±18	229±19	1.2	ribosomal protein L19
*Rpl23*	329±14	442±49	1.3	ribosomal protein L23
*Rpl24*	239±37	327±47	1.3	ribosomal protein L24
*Rpl26*	29±6	44±11	1.5	ribosomal protein L26
*Rpl27*	100±13	153±19	1.5	ribosomal protein L27
*Rpl28*	190±21	275±38	1.4	ribosomal protein L28, (RGD1565183)
*Rpl3*	62±3	90±14	1.4	ribosomal protein L3
*Rpl30*	10±9	37±18	3.6	ribosomal protein L30
*Rpl35*	275±31	360±33	1.3	ribosomal protein L35
*Rpl37a*	75±20	136±19	1.7	ribosomal protein L37a
*Rpl4*	254±31	377±44	1.4	ribosomal protein L4
*Rpl41*	384±24	570±98	1.4	ribosomal protein L41
*Rpl7*	181±20	227±23	1.2	ribosomal protein L7
*Rplp0*	747±27	969±93	1.3	ribosomal protein, large, P0
*Rplp1*	75±20	111±29	1.3	ribosomal protein, large, P1
*Rps10l1*	37±5	59±8	1.5	ribosomal protein S10 (chr6:84968906–84969452)
*Rps11*	302±11	395±37	1.3	ribosomal protein S11
*Rps13*	28±3	41±5	1.4	ribosomal protein S13
*Rps15*	356±28	516±64	1.4	ribosomal protein S15
*Rps15a*	15±0.5	22±3	1.5	ribosomal protein S15a
*Rps16*	254±22	395±57	1.5	ribosomal protein S16
*Rps18*	53±7	76±11	1.4	ribosomal protein S18
*Rps2*	20±6	33±5	1.6	ribosomal protein S2
*Rps23*	74±6	109±12	1.4	ribosomal protein S23
*Rps24*	101±14	139±22	1.4	ribosomal protein S24
*Rps25*	19±3	28±3	1.4	ribosomal protein S25
*Rps27*	380±38	512±86	1.3	ribosomal protein S27
*Rps27a*	141±9	210±44	1.4	ribosomal protein S27a
*Rps27l*	90±10	167±23	1.8	ribosomal protein S27-like
*Rps29*	408±38	470±22	1.1	ribosomal protein S29
*Rps3*	195±11	280±46	1.4	ribosomal protein S3
*Rps5*	445±28	541±22	1.2	ribosomal protein S5
*Rps7*	75±8	106±17	1.4	ribosomal protein S7 (chr5:172581803–172582388)
*Rps7*	35±3	53±8	1.4	ribosomal protein S7(chr6:56601564–56606473)
*Rpsa*	240±14	351±50	1.4	ribosomal protein SA

Genes from the canonical pathway from Ingenuity Pathway Analysis that were significantly affected by olanzapine treatment are shown.

### Canonical and upstream pathway analysis

In addition to the canonical pathways mentioned earlier (e.g., glycolysis, lipid metabolism, EIF-2 signaling/protein synthesis pathway, apoptosis, etc.), bioinformatics implicated other pathways (S6 and S7 Tables) and predicted upstream pathways affected by olanzapine (S8 Table). Both Integrin Linked kinase (ILK Pathway, 43 genes significantly affected, p = 1.3e-16, S6 Table) and Calcium Sensing pathways (39 genes significantly affected, p = 2.3e-14, S7 Table) were affected by olanzapine. Both have been previously linked to muscle fiber transitions.

In terms of potential mechanisms for these effects, we can first consider one involving elevated glucocorticoids (NCBI Geo profile GDS2688) [32]. Olanzapine elevates glucocorticoids [31], and we did observe a number of glucocorticoid response genes up regulated by olanzapine. For example, metallothioneins such as *Mt1m*, *Mt2A* were significantly up regulated as were *Art3*, *Ankrd9*, *Cebpd*, *Dapk1*, *Eif4ebp1*, *Gadd45g*, *IL15*, *Sar1b*, *Serpina3n*, *Slc30a2*, *Sult1a1*, *Thrsp*, *VPS45* (S1 Table). However other genes that are induced by methylprednisolone in skeletal muscle during time course studies) [32], were either not affected (e.g., *Ddit4*, *Trim63*) or down regulated by olanzapine (e.g., *LPL*, *Csrp3*). Furthermore most of the changes we observed in genes encoding proteins or enzymes of the sarcomere, glycolysis, lipid metabolism, amino acid metabolism and protein turnover could not be ascribed to this mechanism. Nevertheless, upstream pathway analysis did implicate a large set of other potential regulators (S8 Table).

The top 20 potential regulators from IPA analysis included RICTOR (inhibited), *MYCN* (activated), *MYC*, insulin and IGF receptors (inhibited), *TP53*, *PPARGC1A* and *PPARG* (inhibited), *HRAS* (activated) and Huntingtin (*HTT*). For example, inhibition of the rapamycin-insensitive companion of mTOR, *RICTOR* (p = 4.9e-52, Activation Z score: -6.3) was predicted. That pathway includes many of the same genes implicated in ILK and EIF-2α signaling mentioned earlier. Additionally, activation was predicted for the Myc and N-myc proto-oncogene transcription factors, *MYC* (p = 2.3e-51, activation Z score: 2), *MYCN* (p = 1.2E-34, activation Z score: 5.2) along with mitogen-activated protein kinase kinase kinase kinase 4 (*Map4k4*, p = 8.2e-15, activation Z score: 3.7). However, some growth factor pathways were predicted to be inhibited, including vascular endothelial growth factor A (*VEGF*, whose expression was also significantly decreased, S2 Table, p = 2e-15 for the pathway, Activation Z score: -2.5), transforming growth factor beta 1 (*TGFB1*, p = 2.1e-15, Activation Z score: -4.3) and Insulin-like growth factor 1 (*IGF1*, p = 1.3e-10, Activation Z score: -3.2). The loss of genes associated with VEGF signaling is consistent with the observation that fast fibers are less vascularized as cited earlier. Several predicted regulators (S8 Table) also suggest an overall “endocrine disruption”. However, there is no known link between any of the above pathways and receptors that olanzapine is known to inhibit.

A potential mechanism for a red to white or a white to “whiter” switch could be increased Calcineurin-A/NFAT signaling associated with the increase in myozenin-1 expression we observed [55]. Myozenin-1 (*MYOZ1*, also called FatZ1) encodes the calsarcin-2 protein. *MYOZ1* expression is restricted to fast skeletal muscle in contrast to myozenin-2 (*MYOZ2*, *FatZ1*) which encodes calsarcin-1, found in adult cardiac and slow-twitch skeletal muscle [55]. Consistently, rat gastrocnemius, which contains a greater number of white than red fibers, had about 72% of its myozenin FPKM values as MYOZ1. MYOZ1 increased, whereas MYOZ2 decreased after olanzapine treatment (Table 1 and S1 Table). Knock-out mouse studies suggest that myozenins act as a brake on calcineurin signaling which plays a critical role in the promotion of fast-twitch fibers [55]. Myoz1 KO mice have similar muscle size, lower body weight and more slow oxidative muscle fibers [42]. Chronic olanzapine treatment causes greater adiposity without a change in body weight implying a loss of lean mass [17, 18], and in this study a greater expression of Myoz1 in skeletal muscle and other evidence of fiber type changing to the fastest twitch type, IIb. Other findings consistent with a role for Calcineurin-A/NFAT signaling/loss of lean mass are the alterations in ILK, Rictor and Calcium Signaling pathways mentioned earlier along with evidence of deceased Insulin receptor and IGF-1 Receptor Signaling pathways observed (S8 Table). Nevertheless, it is unclear how olanzapine would increase myosenin-1. Albeit, this is also the case for a number of the earlier described upstream pathways found to be affected through Ingenuity Pathway Analysis, with the exception of the activation of the corticosteroid response genes due to the rise in glucocorticoids initiated by olanzapine [31].

## Conclusions

In summary, our data indicate that at doses that cause a mild hyperglycemia, much less than that observed acutely with higher doses of the drug [31], olanzapine is eliciting a rapid and dramatic effect on the expression of a host of skeletal muscle genes that appears to be aimed at switching the muscle fiber type along with associated metabolism from a slower (more oxidative) to a faster (more glycolytic) fiber type (Fig 3). Studies have previously shown that olanzapine exerts acute effects leading to increased plasma glucose, impaired hepatic glucose and muscle glucose metabolism while dramatically increasing the use of fat as a fuel [12–14, 18, 31, 69, 70]. It might seem counter intuitive therefore that the response of muscle to that situation would be to switch to a fiber type that was less metabolically flexible and less able to oxidize fat. However in insulin resistant obesity and T2D, there is also an increase of use of fat as a fuel as the muscle becomes unable to transport glucose and in those metabolic states skeletal muscle also undergoes similar fiber type transitions from more oxidative to more glycolytic [24–27]. A muscle fiber type transition could be important in the setting of olanzapine inducing adiposity because such a transition might decrease the basal energy requirements of lean tissue. This in turn could lead to a surfeit of energy that along with other factors promote the adiposity that is observed in humans and animal models treated with olanzapine. In addition to differences in resting energy requirements and fuel selection, fast-twitch glycolytic fibers are also more susceptible to atrophy arising from various metabolic pathologies compared to slow-twitch oxidative fibers [23]. That could also play a role in the shifts in body composition in observed in animal models and humans treated with SGAs, e.g.: [18]. Finally, these findings are important because a slower to faster muscle fiber type transition may be potentially reversible by the type of exercise that is selected. For example, it is known that endurance exercise training can cause the reverse effect, fast to slow twitch transitions [71].

## Supporting Information

S1 Fig Correlation between RNA-Seq and QT-RTPCR data. Comparison of gene expression changes after olanzapine infusion as measured by QT-RTPCR and RNA-Seq.
(TIFF)

S1 Table Selected output and FPKM results from Cufflinks analysis and descriptive statistics. This table contains the following Excel column headers: A—ENSEMBLE Gene ID; B—Official Gene Symbol (gene_short); C—Gene locus (locus, chr1 = chromosome 1 etc.); D—Control animal #3 (FKPM units); E—Olanzapine treated animal #4 (FKPM units); F—Control animal #9 (FKPM units); G—Olanzapine treated animal #10 (FKPM units); H—Control animal #11 (FKPM units); I—Olanzapine treated animal #12 (FKPM values); J—Mean—Control group (C-mean, FKPM units); K—Standard Error of the Mean—Control group (C-SE, FKPM units); L—Mean—Olanzapine group units = FKPM (Ola-mean, FKPM units); M—Standard Error of the Mean—Olanzapine group (Ola-SE, FKPM units); N—Normalized fold change from S2 Table 3; O—p<0.05? True = 1, False = 0 from S2 Table; P—p<0.001? True = 1, False = 0 from S2 Table
(XLSX)

S2 Table Statistical analysis of RNA Seq data, output from DEGseq 1.18.0 R package. This table contains the following Excel column headers: A—ENSEMBLE Gene ID; B—Sum of Olanzapine FPKM values for all three animals; C—Sum of Control FPKM values; D—log_2_ fold change Olanzapine/Control; E—log_2_ (fold change) Normalized; F—p-value comparing olanzapine to control; G—q-value (method 1 Benjamini et al. 1995); H—q-value (method 2 Storey et al. 2003); I—Signature (p-value < 0.001); J—Normalized fold change; K—p<0.05? True = 1, False = 0; L—p<0.001? True = 1, False = 0.
(XLSX)

S3 Table Glycolytic, TCA Cycle and Mitochondrial Shuttle Gene Expression in Gastrocnemius Muscle after Olanzapine Infusion. Table annotated in the file.
(PDF)

S4 Table Olanzapine inhibition of Skeletal Muscle Genes in Branched Chain Amino Acid oxidation. Table annotated in the file.
(PDF)

S5 Table Genes altered by Olanzapine in Canonical Mitochondrial Dysfunction Pathway. Table annotated in the file.
(PDF)

S6 Table Genes altered by Olanzapine in Canonical ILK Pathway. This table contains the following Excel column headers: A—Symbol; B—Entrez Gene Name; C—Ensembl; D—Fold Change; E—Location; F—Type; G—Biomarker Application(s); H—Drug(s); I—Entrez Gene ID for Human; J—Entrez Gene ID for Mouse; K—Entrez Gene ID for Rat
(XLS)

S7 Table Genes altered by Olanzapine in Calcium Signaling Pathway. This table contains the following Excel column headers: A—Symbol; B—Entrez Gene Name; C—Ensembl; D—Fold Change; E—Location; F—Type; G—Biomarker Application(s); H—Drug(s); I—Entrez Gene ID for Human; J—Entrez Gene ID for Mouse; K—Entrez Gene ID for Rat
(XLS)

S8 Table Potential Upstream Regulators. Upstream regulators from Ingenuity Pathway Analysis. The table contains the following Excel column headers: A—Upstream Regulator; B—Fold Change; C—Molecule Type; D—Predicted Activation State; E—Activation z-score; F—p-value of overlap; G—Target molecules in dataset; H—Mechanistic Network
(XLSX)
