# Supplementary figures and images for: RNA Sequencing Reveals a Slow to Fast Muscle Fiber Type Transition after Olanzapine Infusion in Rats
Source: PLoS One. 2015 Apr 20;10(4):e0123966. doi: 10.1371/journal.pone.0123966 (PMC4404103; doi:10.1371/journal.pone.0123966)

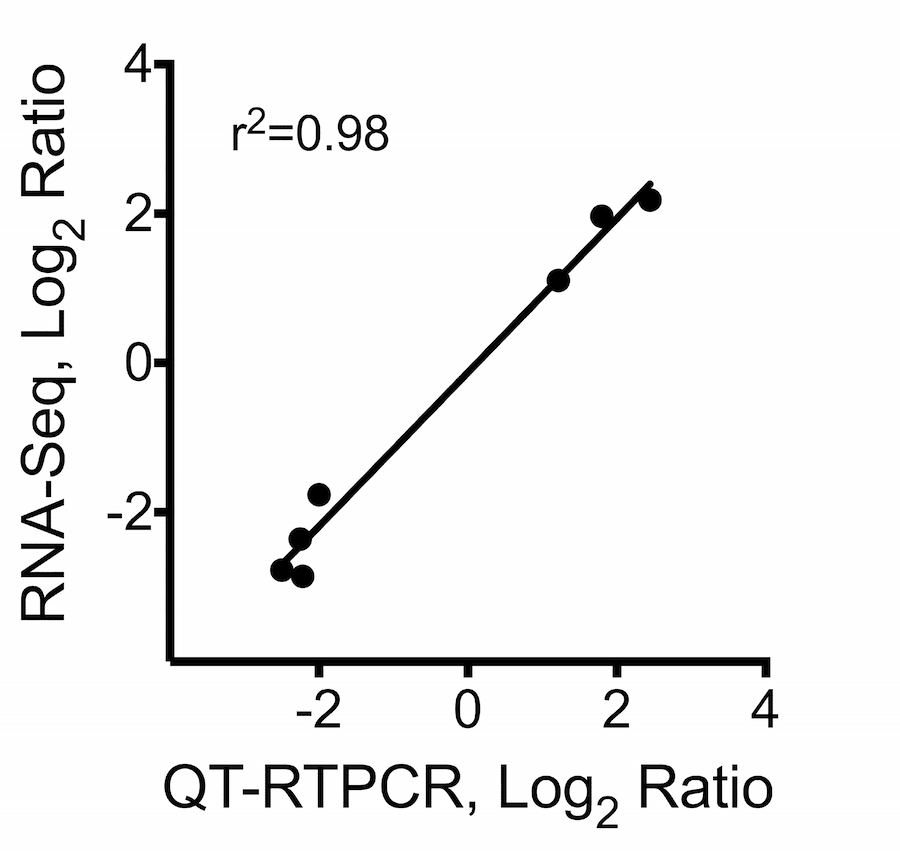

Supplement: S1 Fig — Comparison of gene expression changes after olanzapine infusion as measured by QT-RTPCR and RNA-Seq. (TIFF) [file pone.0123966.s001.tiff]
