# Supplementary material for: RNA Sequencing Reveals a Slow to Fast Muscle Fiber Type Transition after Olanzapine Infusion in Rats
Source: PLoS One. 2015 Apr 20;10(4):e0123966. doi: 10.1371/journal.pone.0123966 (PMC4404103; doi:10.1371/journal.pone.0123966)
Supplement: S3 Table — Table annotated in the file. (PDF) [file pone.0123966.s004.pdf]

**S3 Table. Glycolytic, TCA cycle and mitochondrial shuttle gene expression in gastrocnemius muscle after olanzapine infusion.**

| *Gene Name* | Molecular Identity | Vehicle Control Group, FPKM[a] | Olanzapine Infusion Group, FPKM[a] | Normalized Fold Change |
|---|---|---|---|---|
| *Aco2* | Aconitase 2, mitochondrial | 666±43 | 603±601 | -1.2 |
| *AldoA* | Aldolase A, fructose-bisphosphate | 7535±1059 | 10823±1062 | +1.4 |
| *Aldoart2* | Aldolase 1 A retrogene 2 | 35±7 | 54±5 | +1.6 |
| *Cs* | Citrate Synthase | 299±19 | 239±23 | -1.3 |
| *Dld* | Dihydrolipoamide dehydrogenase; a.k.a.: *Dldh, E3* | 84± 7 | 59±5 | -1.5 |
| *Dlst* | Dihydrolipoamide S-Succinyltransferase (E2 Component Of 2-Oxo-Glutarate Complex) | 118±10 | 92.03±11 | -1.3 |
| *Eno3* | Enolase 3, beta, muscle | 4485±293 | 6423±1012 | +1.4 |
| *Fbp2* | Fructose-1,6-bisphosphatase 2 | 32±3 | 79±25 | +2.5 |
| *Fh* | Fumarate hydratase | 243±22 | 186±14 | -1.4 |
| *Gapdh* | Glyceraldehyde-3-phosphate dehydrogenase | 184±22 | 234±23 | +1.3 |
| *Gpi* * | Glucose-6-phosphate isomerase | 530±45 | 691±87 | +1.3 |
| *Hk2* | Hexokinase 2 | 16±4 | 9±2 | -1.9 |
| *Idh2* | Isocitrate dehydrogenase [NADP], mitochondrial | 750±96 | 340±15 | -2.3 |
| *Idh3a* | Isocitrate dehydrogenase 3 (NAD+) alpha | 190±12 | 223± 32 | 1.1 |
| *Idh3B* * | Isocitrate dehydrogenase 3 (NAD+) beta | 350±36 | 285±12 | -1.3 |
| *Ldha* | Lactate dehydrogenase A (M, fast muscle type) | 180±10 | 268±21 | +1.5 |
| *Ldhb* | Lactate dehydrogenase B (H, heart slow muscle type) | 463±100 | 97±30 | -5.0 |
| *Mdh2* | Malate dehydrogenase 2, NAD (mitochondrial) | 894±50 | 841±85 | -1.1 |
| *Myo1c* [b] | myosin-Ic | 21±1 | 12±2 | -1.7 |
| *Ogdh* | Oxoglutarate (alpha-ketoglutarate) dehydrogenase (lipoamine); a.k.a.: *E1k, Ogdc* | 243±12 | 179±29 | -1.4 |
| *Pgam2* | Phosphoglycerate mutase 2 (muscle) | 3621±165 | 6534±947 | +1.7 |
| *Pgk1* | Phosphoglycerate kinase 1 | 409±30 | 668±67 | +1.6 |
| *Pkm* | Pyruvate kinase, muscle | 88±4 | 136±25 | +1.5 |
| *Sdha* | Succinate dehydrogenase complex, subunit A, flavoprotein (Fp); a.k.a: *Sdhf, Sdh2* | 291±22 | 227±31 | -1.3 |
| *Sdhb* | Succinate dehydrogenase complex, subunit B, iron sulfur (Ip); a.k.a.: *Sdh1, Sdhip, Pgl4* | 606±60 | 478±19 | -1.4 |
| *Sh2b2* [c] | SH2B adaptor protein 2 (also, APS) | 15±6 | 9±0.6 | -1.7 |
| *Slc16a1* | Monocarboxylate transporter 1 (a.k.a.: MCT1, e.g., lactate/pyruvate) | 36±5 | 14±2 | -2.5 |
| *Slc16a7* | [Monocarboxylate transporter 3 (a.k.a.: *MCT3*, e.g., lactate/pyruvate)] | 132±4 | 177±38 | +1.3 |
| *Sorbs1* [c] | SH2B adaptor protein 2; a.k.a.: CAP | 17±2 | 10±2 | -1.7 |
| *Sucla2* | Succinate-CoA ligase, GDP-forming, alpha subunit, a.k.a.: *Sucla1* | 264±31 | 227±13 | -1.2 |
| *Suclg2* | Succinate-CoA ligase, GDP forming, beta subunit | 32±2 | 22±1 | -1.5 |

Data are mean±SE, Normalized Fold Change calculated by DESeq software. Footnotes:
[a]FPKM, Fragments Per Kilobase Of Exon Per Million Fragments Mapped.
[b]Involved in glucose transporter 4 (Slc2A4) recycling in response to insulin.
[c] Involved in insulin signaling to glucose transporter 4 (Slc2A4)
An asterisk (*) indicates one of 164 prioritized obesity or T2D candidate genes from Tiffin et al (Tiffin N, Adie E, Turner F, Brunner HG, van Driel MA, Oti M *et al.* Computational disease gene identification: a concert of methods prioritizes type 2 diabetes and obesity candidate genes. *Nucleic acids research* 2006; **34**(10)**:** 3067-3081).
