# Supplementary material for: RNA Sequencing Reveals a Slow to Fast Muscle Fiber Type Transition after Olanzapine Infusion in Rats
Source: PLoS One. 2015 Apr 20;10(4):e0123966. doi: 10.1371/journal.pone.0123966 (PMC4404103; doi:10.1371/journal.pone.0123966)
Supplement: S4 Table — Table annotated in the file. (PDF) [file pone.0123966.s005.pdf]

S4 Table. Olanzapine inhibition of skeletal muscle genes in branched chain amino acid degradation.

Ensembl	Symbol	Entrez Gene Name	NFC
ENSRNOG00000009845	ACADM	Acyl-CoA dehydrogenase, C-4 to C-12 straight chain	-1.8
ENSRNOG00000020624	ACADSB	Acyl-CoA dehydrogenase, short/branched chain	-1.8
ENSRNOG00000007862	ACAT1	Acetyl-CoA acetyltransferase 1	-1.4
ENSRNOG00000011419	ALDH6A1	Aldehyde dehydrogenase 6 family, member A1	-2.0
ENSRNOG00000020607	BCKDHA *	Branched chain keto acid dehydrogenase E1, alpha polypeptide	-1.4
ENSRNOG00000006364	DLD	Dihydrolipoamide dehydrogenase	-1.5
ENSRNOG00000024629	HADHA	Hydroxyacyl-CoA dehydrogenase/3-ketoacyl-coa thiolase/enoyl-coa hydratase (trifunctional protein), alpha subunit	-1.8
ENSRNOG00000010800	HADHB	Hydroxyacyl-CoA dehydrogenase/3-ketoacyl-coa thiolase/enoyl-coa hydratase (trifunctional protein), beta subunit	-1.4
ENSRNOG00000008063	HIBADH	3-hydroxyisobutyrate dehydrogenase	-1.7
ENSRNOG00000009421	IVD *	Isovaleryl-CoA dehydrogenase	-1.8

Skeletal muscle genes from in branched chain amino acids metabolism that were significantly affected by olanzapine. The mean ± SE and p values are in supplementary tables 1 and 2. NCF, normalized fold change from DESeq analysis of Supplementary Table 1. An asterisk(*) indicates one of 164 prioritized obesity or T2D candidate genes from Tiffin et al (Tiffin N, Adie E, Turner F, Brunner HG, van Driel MA, Oti M *et al.* Computational disease gene identification: a concert of methods prioritizes type 2 diabetes and obesity candidate genes. *Nucleic acids research* 2006; **34**(10)**:** 3067-3081.). URL for reference rat pathway:
http://www.genome.jp/kegg-bin/show_pathway?org_name=rno&mapno=00280&mapscale=&show_description=hide
