# Supplementary material for: RNA Sequencing Reveals a Slow to Fast Muscle Fiber Type Transition after Olanzapine Infusion in Rats
Source: PLoS One. 2015 Apr 20;10(4):e0123966. doi: 10.1371/journal.pone.0123966 (PMC4404103; doi:10.1371/journal.pone.0123966)
Supplement: S5 Table — Table annotated in the file. (PDF) [file pone.0123966.s006.pdf]

S5 Table. Mitochondrial Dysfunction Pathway Analysis

Ensembl ID	Symbol	Entrez Gene Name	NFC
ENSRNOG00000006067	AIFM1	apoptosis-inducing factor, mitochondrion-associated, 1	-1.6
ENSRNOG00000017032	ATP5A1	ATP synthase, H+ transporting, mitochondrial F1 complex, alpha subunit 1, cardiac muscle	-1.2
ENSRNOG00000002840	ATP5B	ATP synthase, H+ transporting, mitochondrial F1 complex, beta polypeptide	-1.2
ENSRNOG00000014625	ATP5D	ATP synthase, H+ transporting, mitochondrial F1 complex, delta subunit	1.1
ENSRNOG00000049912	Atp5e	ATP synthase, H+ transporting, mitochondrial F1 complex, epsilon subunit	1.3
ENSRNOG00000007235	ATP5G1	ATP synthase, H+ transporting, mitochondrial Fo complex, subunit C1 (subunit 9)	1.3
ENSRNOG00000015320	ATP5G2	ATP synthase, H+ transporting, mitochondrial Fo complex, subunit C2 (subunit 9)	1.1
ENSRNOG00000038744	ATP5H	ATP Synthase, H+ Transporting, Mitochondrial Fo Complex, Subunit D	1.4
ENSRNOG00000000064	ATP5I	ATP synthase, H+ transporting, mitochondrial Fo complex, subunit E	1.1
ENSRNOG00000027049	ATP5J2	ATP synthase, H+ transporting, mitochondrial Fo complex, subunit F2	1.2
ENSRNOG00000028884	ATP5L	ATP synthase, H+ transporting, mitochondrial Fo complex, subunit G	1.2
ENSRNOG00000001991	ATP5O	ATP synthase, H+ transporting, mitochondrial F1 complex, O subunit	1.2
ENSRNOG00000008364	CAT	catalase	-1.7
ENSRNOG00000038951	COX17	COX17 cytochrome c oxidase copper chaperone	1.2
ENSRNOG00000018816	COX5A	cytochrome c oxidase subunit Va	1.1
ENSRNOG00000016660	COX5B	cytochrome c oxidase subunit Vb	1.1
ENSRNOG00000024309	COX6B1	cytochrome c oxidase subunit VIb polypeptide 1 (ubiquitous)	1.1
ENSRNOG00000010807	COX6C	cytochrome c oxidase, subunit VIc	1.1
ENSRNOG00000042903	COX7A2	cytochrome c oxidase subunit VIIa polypeptide 2	2.5
ENSRNOG00000004526	COX7A2L	cytochrome c oxidase subunit VIIa polypeptide 2 like	1.2
ENSRNOG00000028451	COX7B	cytochrome c oxidase subunit VIIb	1.1
ENSRNOG00000030237	COX7C	cytochrome c oxidase subunit VIIc	1.2
ENSRNOG00000021177	COX8A	cytochrome c oxidase subunit VIIIA (ubiquitous)	-1.3
ENSRNOG00000014656	Cox8b	cytochrome c oxidase subunit VIIIb	1.1
ENSRNOG00000010438	CPT1B	carnitine palmitoyltransferase 1B (muscle)	-1.5
ENSRNOG00000001420	FIS1	fission 1 (mitochondrial outer membrane) homolog (S. cerevisiae)	1.2
ENSRNOG00000033824	GPD2	glycerol-3-phosphate dehydrogenase 2 (mitochondrial)	1.5
ENSRNOG00000013604	GPX4	glutathione peroxidase 4	1.2
ENSRNOG00000031979	MT-ATP6	ATP synthase F0 subunit 6	-1.2
ENSRNOG00000034234	MT-CO1	cytochrome c oxidase subunit I	-1.4
ENSRNOG00000030371	MT-CO2	cytochrome c oxidase subunit II	-1.2
ENSRNOG00000030700	MT-CO3	cytochrome c oxidase III	-1.2
ENSRNOG00000031766	MT-CYB	cytochrome b	-1.2
ENSRNOG00000030644	MT-ND1	NADH dehydrogenase, subunit 1 (complex I)	-1.3
ENSRNOG00000031033	MT-ND2	MTND2	-1.3
ENSRNOG00000033615	MT-ND3	NADH dehydrogenase, subunit 3 (complex I)	1.3
ENSRNOG00000029707	MT-ND4	NADH dehydrogenase, subunit 4 (complex I)	-1.5
ENSRNOG00000029971	MT-ND5	NADH dehydrogenase, subunit 5 (complex I)	-1.5
ENSRNOG00000029042	MT-ND6	NADH dehydrogenase, subunit 6 (complex I)	-1.4

ENSRNOG00000017571	NDUFA2	NADH dehydrogenase (ubiquinone) 1 alpha subcomplex, 2, 8kDa	1.2
ENSRNOG00000014224	NDUFA3	NADH dehydrogenase (ubiquinone) 1 alpha subcomplex, 3, 9kDa	1.3
ENSRNOG00000005512	NDUFA4	NADH dehydrogenase (ubiquinone) 1 alpha subcomplex, 4, 9kDa	1.1
ENSRNOG00000005698	NDUFA5	NADH dehydrogenase (ubiquinone) 1 alpha subcomplex, 5	-1.1
ENSRNOG00000008569	NDUFA6	NADH dehydrogenase (ubiquinone) 1 alpha subcomplex, 6, 14kDa	1.1
ENSRNOG00000018129	NDUFAB1	NADH dehydrogenase (ubiquinone) 1, alpha/beta subcomplex, 1, 8kDa	1.1
ENSRNOG00000008329	NDUFB11	NADH dehydrogenase (ubiquinone) 1 beta subcomplex, 11, 17.3kDa	1.2
ENSRNOG00000026616	NDUFB2	NADH dehydrogenase (ubiquinone) 1 beta subcomplex, 2, 8kDa	1.3
ENSRNOG00000024539	NDUFB6	NADH dehydrogenase (ubiquinone) 1 beta subcomplex, 6, 17kDa	1.1
ENSRNOG00000014078	NDUFB8	NADH dehydrogenase (ubiquinone) 1 beta subcomplex, 8, 19kDa	1.4
ENSRNOG00000009364	NDUFB9	NADH dehydrogenase (ubiquinone) 1 beta subcomplex, 9, 22kDa	1.2
ENSRNOG00000011849	NDUFS1	NADH dehydrogenase (ubiquinone) Fe-S protein 1, 75kDa (NADH-coenzyme Q reductase)	-1.6
ENSRNOG00000009155	NDUFS3	NADH dehydrogenase (ubiquinone) Fe-S protein 3, 30kDa (NADH-coenzyme Q reductase)	1.3
ENSRNOG00000029339	NDUFS5	NADH dehydrogenase (ubiquinone) Fe-S protein 5	1.2
ENSRNOG00000024568	NDUFS7	NADH dehydrogenase (ubiquinone) Fe-S protein 7, 20kDa (NADH-coenzyme Q reductase)	1.1
ENSRNOG00000017446	NDUFS8	NADH dehydrogenase (ubiquinone) Fe-S protein 8, 23kDa (NADH-coenzyme Q reductase)	1.2
ENSRNOG00000018117	NDUFV1	NADH dehydrogenase (ubiquinone) flavoprotein 1, 51kDa	-1.3
ENSRNOG00000042503	NDUFV2	NADH dehydrogenase (ubiquinone) flavoprotein 2, 24kDa	1.2
ENSRNOG00000027593	NDUFV3	NADH dehydrogenase (ubiquinone) flavoprotein 3, 10kDa	1.2
ENSRNOG00000005130	OGDH	oxoglutarate (alpha-ketoglutarate) dehydrogenase (lipoamide)	-1.4
ENSRNOG00000018289	PARK7	parkinson protein 7	1.5
ENSRNOG00000015385	PINK1	PTEN induced putative kinase 1	-1.2
ENSRNOG00000010958	PRDX3	peroxiredoxin 3	-1.2
ENSRNOG00000020941	PSENEN	presenilin enhancer gamma secretase subunit	1.2
ENSRNOG00000013331	SDHA	succinate dehydrogenase complex, subunit A, flavoprotein (Fp)	-1.3
ENSRNOG00000007967	SDHB	succinate dehydrogenase complex, subunit B, iron sulfur (Ip)	-1.3
ENSRNOG00000019048	SOD2	superoxide dismutase 2, mitochondrial	-1.1
ENSRNOG00000016952	UQCR11	ubiquinol-cytochrome c reductase, complex III subunit XI	1.1
ENSRNOG00000032134	UQCRC1	ubiquinol-cytochrome c reductase core protein I	-1.1
ENSRNOG00000036742	UQCRC2	ubiquinol-cytochrome c reductase core protein II	-1.4
ENSRNOG00000012550	UQCRH	ubiquinol-cytochrome c reductase hinge protein	1.2
ENSRNOG00000006375	VDAC1	voltage-dependent anion channel 1	-1.3
